# Detection of *Escherichia coli*, *Pseudomonas aeruginosa*, *Salmonella paratyphoid B*, and *Shigella dysentery* in live *Bacillus licheniformis* products using propidium monoazide-real-time-quantitative polymerase chain reaction

**DOI:** 10.3389/fmicb.2022.996794

**Published:** 2022-09-08

**Authors:** Xiaoling Zheng, Yinhuan Wang, WanZi Gong, Qianru Cai, Jue Li, Jiequn Wu

**Affiliations:** ^1^National Medical Products Administration (NMPA) Key Laboratory for Testing and Risk Warning of Pharmaceutical Microbiology, Key Laboratory of Drug Contacting Materials Quality Control of Zhejiang Provincial, Zhejiang Institute for Food and Drug Control, Hangzhou, China; ^2^School of Pharmacy, China Pharmaceutical University, Nanjing, China; ^3^Collaborative Innovation Center of Yangtze River Delta Region Green Pharmaceutical, Zhejiang University of Technology, Hangzhou, China

**Keywords:** propidium monoazide, qPCR, probiotics, *Bacillus licheniformis* live capsule, *Salmonella paratyphoid B*, *Shigella dysentery*, *Pseudomonas aeruginosa*, *Escherichia coli*

## Abstract

To eliminate the influences of excipients and interference of dead bacterial DNA on the detection of *Escherichia coli*, *Pseudomonas aeruginosa*, *Salmonella paratyphoid B*, and *Shigella dysentery* in live *Bacillus licheniformis* capsules, a polymerase chain reaction (PCR) method with high sensitivity and specificity was established. By combining bromide with propidium monoazide (PMA) -real-time quantitative PCR (qPCR) with microporous membrane filtration, excipients were removed, the filtrate was collected, and the bacteria were enriched using the centrifugal method. The optimal PMA working concentration, dark incubation time, and exposure time were determined. Specific *E. coli*, *P. aeruginosa*, *S. paratyphoid B*, and *S. dysentery* primers were selected to design different probes and a multiplex qPCR reaction system was established. The PMA-qPCR method was verified using different concentrations of dead and live bacteria. This method is efficient and accurate and can be widely applied to the detection of aforementioned pathogenic bacterial strains in live *Bacillus licheniformis* products.

## Introduction

Probiotic products, such as live *Bacillus licheniformis* capsule, play an important role in improving the intestinal microbiota imbalance and treating gastrointestinal diseases, such as acute and chronic enteritis and diarrhea (Shen et al., 2014; Akker et al., 2020; Collinson et al., 2020; Selvamani et al., 2022). Probiotic products are beneficial for maintaining the intestinal gut microbiota balance and enhancing human immunity (Isolauri et al., 2001; Górska et al., 2019; Wang et al., 2020; Erica et al., 2021; Bahrul et al., 2022). As their quality is directly related to the safety and effectiveness of clinical treatment, probiotic production is strictly controlled. Therefore, rapid and accurate detection of pathogenic bacteria in live bacteria products is key in ensuring the quality of these products.

At present, the international Codex Alimentarius Commission (CAC), the United States Pharmacopoeia (USP), the European Pharmacopoeia (EP), and the Chinese Pharmacopoeia (C. Ph) all require specific bacteria inspection in probiotic products, mainly including the detection of pathogenic microorganisms or opportunistic pathogens, such as *Salmonella* spp. (*Salmonella paratyphoid B*), *Shigella* spp. (*Shigella dysentery*), *Pseudomonas aeruginosa*, and *Escherichia coli*. In accordance with relevant regulations and standards, the detection of the above pathogenic microorganisms or opportunistic pathogens requires preliminary enrichment culture, spread plate culture, and subsequent identification. However, probiotic products contain billions of live bacteria, which can grow in relevant culture media, greatly interfering with the detection of the above pathogenic bacteria. Furthermore, the traditional culture and identification methods are cumbersome and time-consuming, limiting the accurate and efficient detection of the above-mentioned pathogenic bacteria (Wang et al., 2021).

With the development of rapid live bacteria detection technology, the combination of PCR with nucleic acid dyes for the detection of pathogenic live bacteria has become a current research hotspot (Ou et al., 2019; Dias et al., 2020; Yang et al., 2021). Propidium monoazide (PMA) is an insertional fluorescent nucleic acid-binding dye. Under strong light, PMA undergoes an irreversible covalent crosslinking reaction with DNA through the damaged cell membrane of dead cells and inhibits the amplification of the modified DNA template. However, PMA cannot pass through the intact cell membrane of living cells. Therefore, PMA can be combined with qPCR to detect live pathogenic bacteria in probiotic products (Tavernier and Coenye, 2015; Lv et al., 2016; Takahashi et al., 2018; Telli and Doğruer, 2019). This study aimed to achieve the rapid detection of *Salmonella* spp., *Shigella* spp., *P. aeruginosa*, and *E. coli* in live *Bacillus licheniformis* capsule, wherein PMA-qPCR was combined with microporous membrane filtration to remove a large number of excipients in probiotic products and reduce their interference in subsequent bacterial detection. By optimizing the working concentration, dark incubation time, and exposure time of PMA, we established the optimal PMA treatment method. We designed specific primers and probes for the detection of aforementioned four species of bacteria. An efficient and accurate new method for the simultaneous detection of the aforementioned bacteria and live bacterial products was established.

## Materials and methods

### Materials

#### Instruments

The following instruments were used in this study: PMA-LiteTMLED photolysis instrument (Biotium, United States), Viia7 qPCR instrument (ABI, United States), Biodrop ultramicro protein-nucleic acid analyzer (Baoyude, China), ME2002E electronic balance (Mettler Toledo, Switzerland), HFsafe-1200 biosafety cabinet (Shanghai Likang, China), MLS-3781-PC high-pressure steam sterilizer (Sanyo, Japan), 5424 centrifuge (Ibund, Germany), TW20 precision constant temperature water bath (Youlaibo, Germany), and HTY-761 homogenizer (Zhejiang Tailin, China).

#### Reagents and consumables

The following reagents and consumables were used: PMA (batch No. 19P0129), PMAxxTMdye (No. 40069, Biotium, United States), Takara bacterial genome DNA Extraction Kit (No. AI82587a), Probe qPCR Mix (with UNG) (No. AJ12533A, Bao bioengineering, China), primers and probes for *S. paratyphoid B*, *S. dysentery*, *P. aeruginosa*, and *E. coli* (Shenzhen Huada, China), sterile sodium chloride solution (batch No. LP19121709, Qingdao Lanyan Lvjian, China), sterilized water for injection (No. 180922, Anhui Shuanghe, China), aperture 1.2 and 5.0 μm polyether sulfone microporous filter membrane with a diameter of 50 mm (3M, United States), and Fc502 membrane filter (Zhejiang Tailin, China).

For bacterial cell culture the following media were used: tryptose soy agar (TSA, No. 1068135), trypticase soy broth (TSB, No. 1065575), mannitol salt agar (No. 3304001, Guangdong Huankai, China.), and 7.5% sodium chloride broth (No. 20171020, Qingdao Haibo, China). The applicability of the above media is in line with the provisions of C. Ph 2020 edition.

The live *Bacillus licheniformis* capsule contained a live bacterial concentration of 1 × 10^9^ CFU⋅g^–1^ (No. 201906185, Zhejiang Jingxin, China).

#### Experimental strains

The following experimental strains were used: *Salmonella paratyphoid B* (CMCC [B] 50094), *Shigella dysentery* (CICC 23829), *Proteus mirabilis* (CICC 2516); *Enterobacter sakazakii* (CICC 21544), *P. aeruginosa* (CMCC [B] 10104), *E. coli* (CMCC [B] 44102), *Stenotrophomonas maltophilia*, *Ralstonia Pichia*, *Citrobacter freundii* (laboratory preserved strains), *Burkholderia cepacia* (ATCC 25416), *Burkholderia cepacia* (ATCC BAA-245), and *Burkholderia cepacia* (ATCC BAA-247).

### Methods

#### Preparation of bacterial suspensions

##### Preparation of live bacterial suspensions

The standard strains of *Salmonella* spp., *Shigella* spp., *P. aeruginosa, E. coli, P. mirabilis, E. sakazakii*, *S. maltophilia*, *R. Pichia*, *C. freundii*, *B. cepacia*, and *B. neocepacia*, were inoculated into TSB medium. All strains were cultured at 33°C for 24 h. The number of live *Salmonella* spp., *Shigella* spp., *P. aeruginosa*, and *E. coli* bacteria were counted using TSA plates. The cell numbers of the live bacterial suspensions of *Salmonella* spp., *Shigella* spp., *P. aeruginosa*, and *E. coli* were enumerated as 7.0 × 10^8^ CFU (colony forming units) mL^–1^, 5.0 × 10^8^ CFU mL^–1^, 3.6 × 10^8^ CFU mL^–1^, and 8.3 × 10^8^ CFU⋅mL^–1^, respectively.

##### Determination of the optimal lethal heat treatment time

Live suspensions (10 mL) of *Salmonella* spp., *Shigella* spp., *P. aeruginosa*, and *E. coli*, prepared using the method described in section “Preparation of live bacterial suspensions,” were treated in a boiling water bath for different time periods (0, 10, 20, 30, 40, 50, and 60 min), and then immediately placed on ice for cooling. The number of viable bacteria in the samples was obtained using the CFU counts. The best lethal heat treatment time of the strains was defined as the shortest boiling water bath treatment time required for sterile growth on the plate. At all selected time periods, 10, 20, 30, 40, 50, and 60 min, of boiling water bath treatment, no viable bacterial counts were obtained for *Salmonella* spp., *Shigella* spp., *P. aeruginosa*, and *E. coli* species, indicating that these bacterial strains could be killed after 10 min of boiling water bath treatment. Based on these results, 10 min of boiling water bath treatment was selected as the best lethal heat treatment time for further experiments.

##### Preparation of dead bacterial suspensions

Live bacterial suspensions (10 mL) of *Salmonella* spp., *Shigella* spp., *P. aeruginosa*, and *E. coli* prepared using the method described in section “Preparation of live bacterial suspensions,” were treated in a boiling water bath for 10 min, centrifuged at 8,000 r.min^–1^ for 5 min, and the supernatant subsequently discarded. Then, the pellet containing the dead bacterial suspension of *Salmonella* spp., *Shigella* spp., *P. aeruginosa*, and *E. coli* (10^8^ CFU⋅mL^–1^), was resuspended using 10 mL of sterile distilled water.

#### Pre-treatment of live *Bacillus licheniformis* capsule

A total of 30 g of the *Bacillus licheniformis* live capsule content was aseptically weighed, and added into 270 mL sterile sodium chloride solution dilution, homogenized at 3,500 r.min^–1^ for 30 s to obtain an 1:10 test solution (1 × 10^8^ CFU⋅ mL^–1^). Then, 10 mL of the test solution was filtered using a sterile FC502 filter. The following microporous membranes: 0.45 μm mixed cellulose ester membrane, 1.2 μm, 5 μm polyethersulfone membrane, and 3, 5, and 8 μm nylon membranes were used for analysis and comparison. The results demonstrated no viable bacteria in the filtrate filtered using the mixed cellulose ester membrane. The rest of the membranes were weighed and the 5 μm polyethersulfone membrane excipients had the highest rejection rate.

The 1:10 test solution was filtered using a 5 μm polyethersulfone filter membrane that matched the specifications of the FC502 sterile filter. The filter membrane was replaced for every 10 ml of the 1:10 test solution filtered, and the filtrate was collected by centrifugation at 8 000 r. min^–1^ for 5 min. The supernatant was discarded, and the pellet was resuspended in sterile distilled water to a total volume of 500 μL. The resulting *Bacillus licheniformis* live capsule sample suspension (1 × 10^9^ CFU⋅mL^–1^) was the pre-treatment sample.

#### Optimization of the propidium monoazide treatment conditions and establishment of the quadruple quantitative polymerase chain reaction system

##### Optimization of the propidium monoazide concentration

The four live bacterial suspensions prepared in section “Preparation of live bacterial suspensions” were mixed in 1:1:1:1 ratio according to the cell counting results. The same procedure was followed to prepare a mixture of the dead bacterial suspensions prepared in section “Preparation of dead bacterial suspensions.” A total of 500 μL of each of the above live and dead bacterial mixtures was placed, respectively, in separate centrifuge tubes. Then, 500 μL of the live *Bacillus licheniformis* capsule suspension prepared in section “Pre-treatment of live *Bacillus licheniformis* capsule” was added to each of the above centrifuge tubes and mixed. The mixtures were centrifuged at 8,000 r.min^–1^ for 5 min, the supernatant was discarded, and the pellet was resuspended in sterile distilled water to a final volume of 0.5 mL. PMA aqueous solution, at a concentration of 400 μg⋅mL^–1^, was added to each centrifuge tube to prepare solutions with PMA concentrations of 0, 10, 20, 30, 40, 50, 60, 70, 80, 90, 100, and 110 μg⋅mL^–1^, respectively. The above mixtures were mixed, incubated in the dark at 20–25°C for 15 min, and vortexed to mix every 5 min. Then, they were placed in the centrifuge tube in the photolysis device for 20 min exposure and mixed every 5 min during exposure.

##### Quadruple quantitative polymerase chain reaction system

DNA from bacterial suspensions was isolated using the TaKaRa minibest bacterial genomic DNA extraction kit (TaKaRa, Dalian, China), following the manufacturer’s instructions. Then DNA was treated with PMA as described in section “Optimization of the propidium monoazide concentration.” Subsequently, qPCR detection was conducted to obtain the quantification cycle (Cq) values. The reaction mixture of quadruple qPCR (50 μL) contained: 25 μL of Probe qPCR Mix (with UNG), 1 μL of 0.2 μM upstream primers, 1 μL of 0.2 μM downstream primers, 2 μL of 0.4 μM probe, 5 μL of template DNA, and 4 μL of ddH_2_O. The primers and probes used in the reaction are shown in Table 1. The reaction conditions were as follows: pre-heating at 25°C for 10 min, pre-denaturation at 95°C for 30 s, followed by the PCR reaction: denaturation at 95 °C for 5 s, and annealing extension at 60°C for 34 s (the fluorescence signal was collected in this step), for 40 cycles. A reaction performed with DNA-free water without template control was included as a negative PCR control. Moreover, 16S rRNA was added as internal amplification control (IAC) in each PCR reaction to avoid false negative results.

**TABLE 1:** Primers and probes used in the quadruple quantitative polymerase chain reaction.

Primer	Sequence
*Salmonella paratyphoid B* upstream primer SM-F	5′-GCGGCGTTGGAGAGTGATA-3′
*Salmonella paratyphoid B* downstream primer SM-R	5′-AGCAATGGAAAAAGCAGGATG-3′
*Salmonella paratyphoid B* probe SM-probe	5′-VIC-CATTTCTTAAACGGCGGTGTCTTTCCCT-TAMRA-3′
*Shigella dysentery* upstream primer ZH-F	5′-CGCAATACCTCCGGATTCC-3′
*Shigella dysentery* downstream primer ZH-R	5′-TCCGCAGAGGCACTGAGTT-3′
*Shigella dysentery* probe ZH-probe	5′-FAM-AACAGGTCGCTGCATGGCTGGAA-TAMRA-3′
*Pseudomonas aeruginosa* upstream primer TL-F	5′-AGCAGCCACTCCAAAGAAACC-3′
*Pseudomonas aeruginosa* downstream primer TL-R	5′-CCAGAGCTTCGTCAGCCTTG-3′
*Pseudomonas aeruginosa* probe TL-probe	5′-ROX-TCTGACCGCTACCGAAGACGCAGC-BHQ2-3′
*Escherichia coli* upstream primer DC-F	5′-CAACGAACTGAACTGGCAGA-3′
*Escherichia coli* downstream primer DC-R	5′-CATTACGCTGCGATGGAT-3′
*Escherichia coli* probe DC-probe	5′-Cy5-CCCGCCGGGAATGGTGATTAC-BHQ2-3′

##### Optimization of the propidium monoazide dark incubation time

The bacterial suspensions prepared in section “Preparation of dead bacterial suspensions” were mixed in 1:1:1:1 ratio according to the cell counting results. A total of 500 μL of each of the above live and dead bacterial mixtures were placed, respectively, in separate centrifuge tubes. Then, 500 μL of the live *Bacillus licheniformis* capsule prepared in section “Pre-treatment of live *Bacillus licheniformis* capsule” was added to each centrifuge tube and then mixed. The mixtures were centrifuged at 8,000 r.min^–1^ for 5 min, the supernatant was discarded, and the pellet was resuspended in sterile distilled water to a final volume of 0.5 mL. PMA aqueous solution, at a concentration of 400 μg⋅mL^–1^, was added to each centrifuge tube to a final PMA concentration of 40 μg⋅mL^–1^. The above mixtures were combined, incubated in the dark at 20–25°C, for 0, 5, 10, 15, 20, 25, and 30 min, vortexed to mix every 5 min, placed in the centrifuge tube in the photolysis device for 20 min exposure, and mixed every 5 min during exposure. Subsequently, DNA was extracted according to the method described in section “Quadruple quantitative polymerase chain reaction system” and then analyzed using qPCR.

##### Optimization of the propidium monoazide exposure time

The samples were prepared according to the method described in “Optimization of the propidium monoazide dark incubation time” with a PMA concentration of 40 μg⋅mL^–1^. The samples were mixed, incubated in the dark at 20–25°C for 10 min, vortexed to mix every 5 min, placed in the centrifuge tube in the photolysis device for 0, 5, 10, 15, 20, 25, and 30 min exposure, and mixed every 5 min during exposure. Subsequently, DNA was extracted according to the method described in section “Quadruple quantitative polymerase chain reaction system” and then analyzed using qPCR.

#### Validation of dead and live bacteria at different concentrations

The four viable bacterial suspensions prepared in section “Preparation of live bacterial suspensions”, were mixed at a ratio of approximately 1:1:1:1 based on the counting results. Then, the same procedure was repeated to prepare a mixture of the dead bacterial suspensions as prepared in section “Preparation of dead bacterial suspensions.” The mixtures of the live and dead bacterial suspensions were mixed in proportion, and the proportions of live bacteria in the prepared mixed bacterial suspension were 0, 1, 10, 25, 50, and 100%. Then, 500 μL of the mixed bacterial suspension was placed into different 1.5 mL centrifuge tubes, and the *Bacillus licheniformis* capsule sample suspension was added according to the method described in section “Optimization of the propidium monoazide concentration” to a final volume of 0.5 mL. PMA aqueous solution, at a concentration of 400 μg⋅mL^–1^, was added to each centrifuge tube to a final PMA concentration of 40 μg⋅mL^–1^. The above mixtures were mixed, incubated in the dark at 20–25°C for 10 min, vortexed to mix every 5 min, and placed in the centrifuge tube in the photolysis device for 20 min exposure, and mixed every 5 min. Subsequently, DNA was extracted according to the method described in section “Quadruple quantitative polymerase chain reaction system” and then analyzed using qPCR. No PMA added was used as control.

#### Sensitivity study

The *Salmonella* spp. live bacterial suspension at an initial concentration of 10^8^ CFU⋅mL^–1^, was centrifuged and concentrated ten times. A concentration gradient was prepared by serially diluting the bacterial suspensions (10^7^, 10^6^, 10^5^, 10^4^, 10^3^, 10^2^, and 1 CFU/mL) by 10-fold. A total of 100 μL of each of the bacterial suspension concentrations were used in eight reaction systems, respectively. The *E. coli*, *P. aeruginosa*, and *Shigella* spp. bacterial suspensions prepared in “Preparation of live bacterial suspensions” were mixed, centrifuged, and concentrated ten times, and 100 μL of each suspension was added to the above reaction system. Next, 300 μL of the pre-treated live *Bacillus licheniformis* bacterial capsule sample was added to the reaction system. Subsequently, the samples were treated with PMA at a working concentration of 40 μg⋅mL^–1^, incubated in the dark for 10 min, and exposed for 15 min. DNA was extracted as described in section “Quadruple quantitative polymerase chain reaction system” and then analyzed using qPCR. The sensitivity test methods for *E. coli*, *P. aeruginosa*, and *Shigella* spp. were the same as detailed above.

#### Specificity study

A total of 1 mL of the *Salmonella* spp., *Shigella* spp., *P. aeruginosa*, *E. coli*, *P. mirabilis*, *E. sakazakii*, *S. maltophilia*, *R. pickettii*, *C. freundii*, *B. cepacia*, *B. neocepacia*, and *B. polyphaga* suspensions were added in separate 2 mL centrifuge tubes. Then, the *Bacillus licheniformis* capsule sample suspension was added following the method described in “Optimization of the propidium monoazide concentration” to a final volume of 0.5 mL. The optimal conditions of PMA treatment, extraction of genomic DNA, qPCR reaction, primer probes, and reaction conditions were used as the determined in “Optimization of the propidium monoazide treatment conditions and establishment of the quadruple quantitative polymerase chain reaction system.”

#### Statistical analysis

The obtained data are expressed as $\bar{\chi }$ ± s. One-way analysis of variance was performed using the SPSS 19.0 software. Results are considered statistically significant at *P*-values < 0.05.

## Results and analysis

### Optimization of the propidium monoazide concentration

**FIGURE 1:**
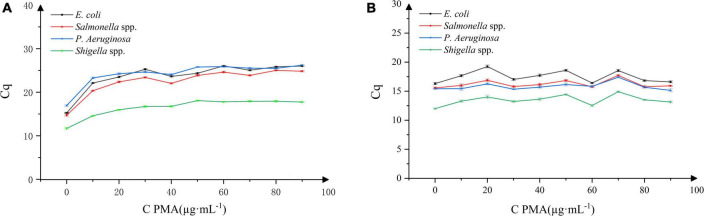
Effect of propidium monoazide (PMA) concentration on DNA amplification in four bacterial strains. *n* = 30, χ ± s; **(A)** dead bacterial strains, **(B)** live bacterial strains.

As shown in Figure 1A, it was evident that with the increasing concentration of PMA, the DNA amplification inhibition rate of the four dead bacterial strains gradually increased. When the concentration of PMA was 40 μg⋅mL^–1^, the Cq values of *E. coli*, *Salmonella*, *P. aeruginosa*, and *Shigella* were 23.628, 22.037, 24.060, and 16.7602, respectively. With the increase in PMA concentration, the Cq value did not changed significantly (*P* > 0.05). As shown in Figure 1B, within the range of PMA concentration selected in this experiment, PMA had no significant effect on the PCR amplification of the DNA extracted from the four live bacterial strains.

Therefore, upon adding PMA at 40 μg⋅mL^–1^ concentration, the binding of PMA to the dead *Bacillus licheniformis* DNA in the sample can be estimated, and the DNA amplification of dead *E. coli*, *Salmonella*, *P. aeruginosa*, and *Shigella* bacteria can be effectively inhibited. At the same time, PMA did not affect the DNA amplification of the above-mentioned live bacterial strains. Thus, 40 μg⋅mL^–1^ was selected as the optimal working concentration of PMA.

### Optimization of the propidium monoazide dark incubation time and exposure time

**FIGURE 2:**
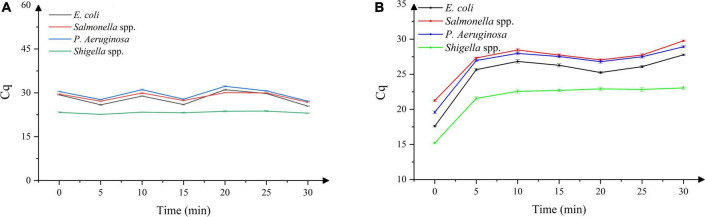
**(A)** Effect of dark incubation time on DNA amplification in four dead bacterial strains, **(B)** effect of exposure time on DNA amplification in four dead bacterial strains. *n* = 21, χ ± s.

As shown in Figure 2A, with the increase in the dark incubation time, the Cq value did not altered significantly. Considering the durability of the method, 10 min was selected as the dark incubation time. As shown in Figure 2B, with the increase in exposure time, the Cq value gradually increased and entered the plateau phase when the exposure time increased to 5 min. Considering the durability of the method and the presence of a certain number of dead bacteria in the *Bacillus licheniformis* capsule samples, 15 min was selected as the exposure time to ensure the effective combination of PMA and dead bacteria.

### Validation of dead and live bacteria at different concentrations

**FIGURE 3:**
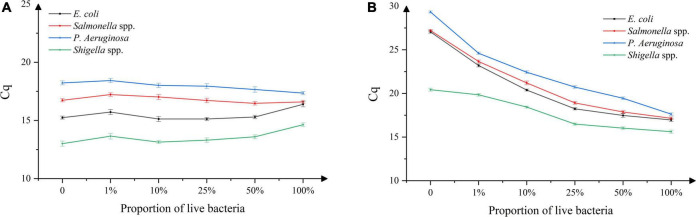
DNA amplification in mixed samples of live/dead bacteria, **(A)** absence of propidium monoazide (PMA), **(B)** presence of propidium monoazide (PMA). *n* = 18, χ ± s.

As shown in Figure 3A, the Cq value in the experimental group without PMA remained unchanged with the increase in the viable bacterial ratios. As shown in Figure 3B, the Cq value of the experimental group with added PMA decreased with the increase in the viable bacterial ratio. In addition, the Cq value was higher than that of the sample without PMA treatment when the viable bacterial ratio was the same. When the proportion of viable bacteria was 100%, there was no difference in Cq values between the samples treated with or without PMA. This indicated that using the experimental conditions established, PMA could effectively bind to the DNA of dead bacteria, thereby inhibiting its subsequent DNA amplification, and avoiding false-positive results. At the same time, PMA had no effect on the detection of viable bacteria, avoiding false-negative results.

### Sensitivity study

**FIGURE 4:**
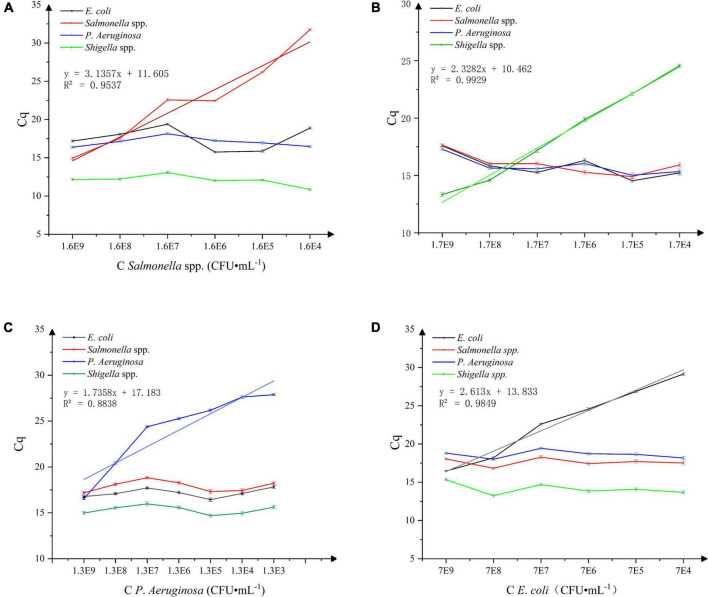
**(A)** Sensitivity of *Salmonella* spp. detection, *n* = 18, χ ± s. **(B)** Sensitivity of *Shigella* spp. detection, *n* = 18, χ ± s. **(C)** Sensitivity of *P. aeruginosa* detection. *n* = 24, χ ± s. **(D)** Sensitivity of *E. coli* detection. *n* = 18, χ ± s.

The results of the *Salmonella* spp. sensitivity study are shown in Figure 4A. A good linear relationship between the Cq values and concentration of *Salmonella* spp. in the live bacterial suspension (1.6 × 10^4^∼1 × 10^9^ CFU⋅mL^–1^) was obtained. With the standard curve equation: y = 3.1357x + 11.605 and *R* = 0.9537, the detection limit was estimated at 1.6 × 10^4^ CFU⋅mL^–1^. The results of the *Shigella* spp. sensitivity study are shown in Figure 4B. A good linear relationship between the Cq values and concentration of *Shigella* spp. in the live bacterial suspension (1.7 × 10^4^∼1 × 10^9^ CFU⋅mL^–1^) was obtained. With the standard curve equation: *y* = 2.3282x + 10.462 and *R* = 0.9929, the detection limit was estimated at 1.7 × 10^4^ CFU⋅mL^–1^. The results of the *P. aeruginosa* sensitivity study are shown in Figure 4C. A good linear relationship between the Cq values and concentration of *P. aeruginosa* in the live bacterial suspension (1.3 × 10^2^∼1 × 10^9^ CFU⋅mL^–1^) was obtained. With the standard curve equation: y = 1.7358x + 17.183 and *R* = 0.8838, the detection limit was estimated at 1.3 × 10^2^ CFU⋅mL^–1^. The results of the *E. coli* sensitivity study are shown in Figure 4D. A good linear relationship between the Cq values and concentration of *E. coli* live bacterial suspension (7.0 × 10^4^∼7.0 × 10^9^ CFU⋅mL^–1^) was obtained. With the standard curve equation: *y* = 2.613x + 13.833 and *R* = 0.9849, the detection limit was estimated at 7.0 × 10^4^ CFU⋅mL^–1^.

### Specificity study

**FIGURE 5:**
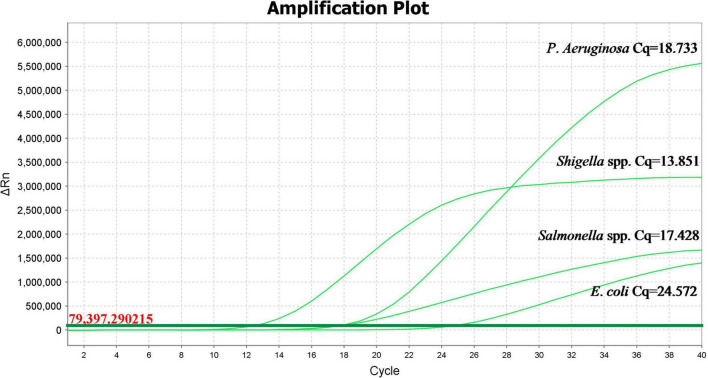
Propidium monoazide-quantitative polymerase chain reaction amplification curves in different bacterial strains.

As shown in Figure 5, *Salmonella* spp. (Cq value: 16.015), *Shigella* spp. (Cq value: 13.951), *P. aeruginosa* (Cq value: 18.099), and *E. coli* (Cq value: 18.125) had an obvious exponentially higher fluorescence amplification signals. *P. mirabilis*, *E. sakazakii*, *S. maltophilia*, *R. pickettii*, *C. freundii*, *B. cepacia*, *B. neocepacia*, and *B. polyphaga* showed no obvious fluorescence amplification curve, indicating that the specificity of this method for the detection of *Salmonella* spp., *Shigella* spp., *P. aeruginosa*, and *E. coli* is higher compared to other tested bacteria.

## Discussion

At present, the combination of PMA with qPCR is mainly used for the detection of common food and clinical pathogens (Truchado et al., 2016; Takahashi et al., 2018; Dorn-In et al., 2019; Zhou et al., 2019). However, the detection of active pathogens in microecological live bacteria products is still in the exploratory stage, especially with PMA combined with multiplex qPCR.

In the process of exploring the establishment of the PMA-qPCR method to detect the four strains of *Bacillus licheniformis* capsules, we also emphasized on studying the effects of various other factors. We optimized the levels of four major factors: turbidity, dark incubation time and exposure-response, salt concentration, and percentage of dead cells in the samples. Firstly, when the turbidity of samples was too high, the light transmittance was reduced, causing incomplete photoreaction of PMA and weakening the effect of PMA on DNA modification easily (Wagner et al., 2008). In this study, the excipients in the samples were removed by microporous membrane filtration to reduce the turbidity of the samples and improve the accuracy of the PMA-qPCR detection method. Secondly, *via* specific penetration through the damaged cell membrane of dead cells, PMA intercalated into DNA in the dark. After that, under the irradiation of strong light, the azide group of the PMA molecule changed into the nitrene groups to form a covalent bond with DNA; free PMA that was not bound to DNA underwent photoreaction with water molecules and became inactive in solution (Soejima et al., 2007, 2008; Feng et al., 2008). Sufficient dark incubation time and exposure time not only ensured that PMA was fully embedded in the DNA double-strand of dead cells and fully bound to DNA, but also that the free PMA was completely inactivated without affecting subsequent reactions. In this study, through experimental exploration, 10 min was finally selected as the dark incubation time, considering the durability of the method and the fact that the *Bacillus licheniformis* capsule sample contained a large number of dead bacteria. During the exposure time, the heat generated by the light would damage the cell membrane of the living bacteria and increase the risk of the cytotoxic effect of PMA. Therefore, 15 min was selected as the exposure time. Thirdly, studies have shown that the presence of salt ions such as sodium chloride could increase the osmotic pressure of the solution, making it easier for PMA to penetrate the cell membrane and reach the interior of the cell. However, Barth et al. (2012) reported high concentrations (>5%) of sodium chloride (NaCl) prevented PMA from inhibiting DNA amplification from dead cells. Therefore, in this study, the addition of 0.85% sodium chloride was chosen to increase the osmotic pressure of the solution. Lastly, Pan’s study (Pan and Breidt, 2007) on *Listeria monocytogenes* found that when the number of dead/live bacteria exceeded 10^4^ CFU⋅mL^–1^or the number of viable bacteria was less than 10^3^ CFU⋅mL^–1^, the Cq value had no linear relationship with the log value of the number of viable bacteria in the samples. Moreover, Løvdal et al. (2011) reported that when the ratio of dead/live bacteria in the experimental samples was less than 10^4^ CFU⋅mL^–1^, the samples treated with PMA could effectively inhibit the amplification of DNA. The research object of this experiment was *Bacillus licheniformis* capsule products, in which the ratio of dead/live bacteria was not more than 10 generally. Under the PMA-qPCR detection conditions established in this study, a good linear relation was obtained, and the amplification DNA of dead bacteria was effectively suppressed.

In the multiple qPCR method established in this study, the detection limit of *Salmonella* spp. was estimated at 1.6 × 10^4^ CFU⋅mL^–1^, of *P. aeruginosa* at 1.3 × 10^2^ CFU⋅mL^–1^, of *E. coli* at 7.0 × 10^4^ CFU⋅mL^–1^, of *Shigella* spp. at 1.7 × 10^4^ CFU⋅mL^–1^. The detection limit of *Salmonella* spp. was consistent with the detection limit of *Salmonella typhimurium* and *Salmonella Sonnei* obtained in the single-plex SD-PMA-PCR and quadruple SD-PMA-PCR studied by Wang et al. (2015), and the detection limit of *P. aeruginosa* was consistent with the research results obtained by Gensberger et al. (2014). However, there was a certain difference between the detection limit of *E. coli* and Eva There’s Gensberger’s research (Gensberger et al., 2014) result of 10^2^–10^3^ CFU⋅mL^–1^, which may be due to the use of water as their research object. There were few matrix interferences in the samples, and less DNA loss in the extraction and purification process, thereby increasing the sensitivity for the detection of *E. coli* in this method. Wang et al. (2015) used PMA-mPCR technology to detect *Shigella* in milk, and attained the detection limit as low as 10^1^ CFU⋅mL^–1^. The difference may be due to the use of liquid as the sample object, owing to much less interference from excipients. The interference of the sample matrix would affect the DNA extraction, PMA and DNA binding efficiency, thus sample preparation should get appropriate attention when optimizing the method.

Overall, the method proposed in this study is efficient, fast, real-time, and accurate. The whole process of screening and identification only takes 3 h, overcoming the problems of traditional methods, wherein the fast and accurate detection of the above-mentioned pathogenic bacteria in microecological live bacteria products remain an unresolved challenge. Thus, the method proposed in this study harbors a wide range of application prospects.

## Data availability statement

The raw data supporting the conclusions of this article will be made available by the authors, without undue reservation.

## Author contributions

XZ, JW, and YW conceived and designed the experiments. JL and QC contributed to reagents, materials, and analysis tools. XZ and YW performed the experiments. JW, XZ, YW, and WG wrote the manuscript. All authors analyzed the data, contributed to the article, and approved the submitted version.
